# Epidemiology of multidrug-resistant *Klebsiella pneumoniae* infection in clinical setting in South-Eastern Asia: a systematic review and meta-analysis

**DOI:** 10.1186/s13756-023-01346-5

**Published:** 2023-12-07

**Authors:** Adamu Salawudeen, Yakubu Egigogo Raji, Garba Gidandawa Jibo, Mohd Nasir Mohd Desa, Hui-min Neoh, Siti Norbaya Masri, Sabrina Di Gregorio, Tengku Zetty Maztura Tengku Jamaluddin

**Affiliations:** 1 Department of Medical Microbiology, Faculty of Medicine and Health Sciences, Universiti Putra Malaysia, 43400 Serdang, Malaysia; 2 Department of Microbiology, Faculty of Science, Gombe State University, Tudun Wada, Gombe, 760214 Gombe State Nigeria; 3 Department of Microbiology, Faculty of Life Sciences, Kebbi State University of Science and Technology, Aliero, 863104 Nigeria; 4 Department of Biomedical Sciences, Faculty of Medicine and Health Sciences, Universiti Putra Malaysia, 43400 Serdang, Malaysia; 5 UKM Medical Molecular Biology Institute (UMBI), Universiti Kebangsaan Malaysia, Jalan Ya’acob Latiff, Bandar Tun Razak, 56000 Kuala Lumpur, Malaysia; 6 Department of Pathology, Clinical Microbiology Unit, College of Health Sciences, Ibrahim Badamasi Babangida University Lapai, Lapai, 911101 Nigeria; 7 Instituto de Investigaciones en Bacteriologia y Virologia Molecular (IBaViM), Facultad de Farmacia y Bioquimica, Universidad de Buenos Aires, Postal Code 1113 Buenos Aires, Argentina; 8 CONICET, National Scientific and Technical Research Council - Argentina, Postal Code 1425 Buenos Aires, Argentina; 9 Infection Control Unit, Department of Medical Microbiology, Hospital Sultan Abdul Aziz Shah, Universiti Putra Malaysia, 43400 Serdang, Malaysia; 10 Department of Medical Microbiology, Hospital Sultan Abdul Aziz Shah, Universiti Putra Malaysia, 43400 Serdang, Malaysia

**Keywords:** Epidemiology, Multidrug-resistant, *Klebsiella pneumoniae*, Infection, Clinical, Setting, Systematic and meta-analysis

## Abstract

The rising prevalence of multidrug-resistant (MDR) and extended-spectrum beta lactamase-resistant (ESBL) *Klebsiella pneumoniae* (*K. pneumoniae*) is an important global public health challenge. This threat is even more pertinent in clinical settings. Morbidity and mortality associated with this condition are alarming particularly in the developing regions of the world. A comprehensive evaluation of the epidemiology of this phenomenon will assist towards the global effort of reducing its burden. So, this systematic review and meta-analysis was conducted to evaluate the epidemiology of MDR *K. pneumoniae* in South-Eastern Asia (SEA). The study was done under the PRISMA guidelines and was preceded by the development of a *priori* protocol. The protocol was then registered in PROSPERO—the public registry for systematic reviews. Seven important outcomes which include the assessment of the overall MDR *K. pneumoniae* prevalence were designed to be evaluated. A literature search was carried out in five selected electronic databases and 4389 were screened. Of these articles, 21 studies that met the eligibility criteria were included in the review. Relevant data were extracted from the included studies. By conducting a quality effect meta-analysis, the pooled prevalence for MDR and ESBL *K. pneumoniae* in SEA was estimated at 55% (CI 9–96) and 27% (CI 32–100) respectively. The review also identified ESBL genes types of allodemic situations occurring mostly in respiratory tract infections. The high prevalence of MDR and ESBL *K. pneumoniae* in this subregion is highly significant and of both public health and clinical relevance. Overall, the findings of this review will assist in the effective prevention and control of this threat in SEA.

**Supplementary Information:**

The online version contains supplementary material available at 10.1186/s13756-023-01346-5.

## Introduction

There are emerging issues of global health importance today and one of them is the rapid spread of multidrug-resistant (MDR) bacteria. The MDR threat is often associated with identified high mortality, prolonged illness, decreased effectiveness of drugs, an easy target for immunocompromised conditions, and high medical cost as the problems associated with multidrug-resistant bacteria [34]. *K. pneumoniae* in particular exhibits the quality of multidrug resistance which enables them to resist last-line antimicrobial medicines such as colistin, tigecycline, and carbapenems increasingly. This particular bacterium can colonize different human systems such as the gastrointestinal tract, nasopharynx, and skin and cause both hospital and community infections [26]. *K. pneumoniae* has been prioritized as one of the top three pathogens of international concern in 2017 by the World Health Organization being a clinically important pathogen causing various infections such as urinary tract infections, respiratory infections, bacteremia, and pneumonia etc. [38]. MDR *K. pneumoniae* involves both extended spectrum beta-lactamases (ESBLs) and carbapenemases. ESBLs are defined as plasmid-mediated and chromosomal enzymes that hydrolyze or inactivate beta-lactam antibiotics or in other words, are enzymes that hydrolyze a wide variety of beta-lactam antibiotics including oxymino-cephalosporins and aztreonam but inhibited by beta-lactam inhibitors like clavulanic acid, tazobactam, and sulbactam [1]. ESBL is produced by a wide range of bacteria classes, but mostly the Enterobacteriaceae family. Infections due to MDR ESBL *K. pneumoniae* have been on the rise in the hospital circle since the discovery of the first ESBL in Germany in 1983 and the south-eastern Asia region is not exempted from this epidemic. ESBL *K. pneumoniae* is a serious public health concern globally. ESBL-producing *K. pneumoniae* strains have been associated with severe outbreak situations and nosocomial infections worldwide. The World Health Organization has estimated the loss of about ten million lives annually and economic output worthy of one hundred trillion USD is in danger due to an increase in diseases caused by drug-resistant organisms [24]. *K. pneumoniae* is understood to be a growing threat in the SEA and very scanty studies are available to address the aforementioned challenges caused by the organisms [5, 25, 38]. Therefore, there is a need for a systematic review and meta-analysis to evaluate the epidemiology of MDR in *K. pneumoniae* infection in a hospital setting of the study region. This review was conducted to examine the prevalence of multidrug-resistant (MDR) *K. pneumoniae* as well as the prevalence of Extended Spectrum Beta-Lactamases (ESBL) *K. pneumoniae* in the clinical/hospital settings of the study regions. The infection should have occurred in the hospital or clinical settings at least 48 h following admission.

## Methods

### Study design

This study was conducted in line with the Preferred Reporting Items for Systematic Review and Meta-Analysis (PRISMA) 2020 guidelines (Additional file 1: S1 File). A preceding protocol (Additional file 1: S3 File) was developed for this systematic review and meta-analysis (SR&MA) according to the PRISMA Protocol (PRISMA-P) guidelines (Additional file 1: S2 File) [23]. The protocol was then registered on the National Institute for Health Research International Prospective Register of Systematic Reviews (PROSPERO 2022, CRD42022299659). Available from: https://www.crd.york.ac.uk/prospero/display_record.php?ID=CRD42022299659.

### Eligibility criteria

The eligibility criteria for this SR&MA were defined as follows:

Inclusion criteria:

- Study type: all observational studies (cross-sectional, cohort, case–control, prevalence surveys) that studied cases of MDR in *K. pneumoniae.*
- Studies conducted in humans among hospital patients.
- Study location: studies conducted in SEA countries.
- Period: there was no time limitation placed on the period of publication included.
- Age and sex: no restriction on studies included.
- Language of publication: only studies published in English language.
- Publication type: both peer-reviewed and preprint articles.

Exclusion criteria:

- Studies involving healthcare workers' (occupational or work-related) infections.
- Studies of community-acquired infections or conducted outside clinical/hospital settings.
- Studies of MDR in animals.
- Studies conducted in countries outside SEA.
- Studies of MDR conducted in other bacteria.
- Studies of a drug (single drug) resistance in *K. pneumoniae.*
- In silico*, *In vitro*,* as well as In vivo (using animal models) studies.
- Studies with incomplete data.
- Letters, books, book chapters, dissertations, review articles, opinion papers, reports, and conference papers.

### Outcomes

*Primary outcome:*

- To determine the overall proportion (prevalence) of MDR *K. pneumoniae* among patients in hospitals.

*Secondary outcomes:*

- To determine ESBL prevalence.
- To assess the predominant ESBL occurring genes.
- To assess ESBL harbouring genes.
- To determine sex distribution of ESBL prevalence.
- To assess the sites of infection for MDR ESBL *K. pneumoniae*
- screening and confirmatory methods adopted in hospitals for the isolation of MDR ESBL *K. pneumoniae*.

### Search and selection strategy

A pre-specified search approach with precise search—terms were developed and used to search five selected electronic bibliographic databases in December 2021. The approach also comprised a grey literature search by searching references of selected (review) articles and conference proceedings. Furthermore, an internet search was carried out on Google Scholar and Google search using specific terms.

### Databases

The selected searched databases include Scopus, MEDLINE, PubMed, CINHAL, and Global Index Medicus (ASEAN Region). The details of specific database searches are provided in the study protocol (Additional file 1: S3 file). Nonetheless, the search algorithm used in the Scopus database is given as follows; (“Epidemiology” OR “Prevalence” OR “Occurrence” OR “Incidence” AND “Multidrug resistant” OR “Multiple drug resistant” OR “Multi-drug resistant” OR “MDR” OR “ESBL” OR “Extended Spectrum Beta Lactamase” OR “Carbapenemase” AND “Klebsiella pneumoniae” OR “K. pneumoniae” OR “Klebsiella infection” AND “Clinical infection” OR “Clinical isolates” OR “Clinical samples” OR “Hospital infection” OR “Hospital-associated infection” OR “Hospital acquired infection” OR “Nosocomial infection” OR “HAI” AND “Indonesia” OR “Cambodia” OR “Vietnam” OR “Singapore” OR “Thailand” OR “Malaysia” OR “PDR Lao” OR “Philippines” OR “Myanmar” OR “Burma” OR “Brunei” OR “Timor-Leste” OR “East Timor”).

### Data management and selection process

The total citations found from the electronic database search (search results) were exported to the reference manager software Mendeley where duplicates were removed (Additional file 1: S4 file). The de-duplicated citations were then exported to the Rayyan Intelligent Systematic Review software `[28]. On the Rayyan software, title/abstract and full-text screening was carried out based on the study inclusion and exclusion criteria. The entire screening process of the review was done by four (4) independent reviewers. Two other reviewers decided on areas of dispute between the four reviewers.

### Data collection process

Extraction of data was conducted after the full-text screening. Some of the relevant data extracted include: (1) study characteristics: title, author, country of study, year of publication, and study design; (2) baseline characteristics of study population: sample size, site of infection; (3) the proportion of MDR *K. pneumoniae*, ESBL *K. pneumoniae*, predominant ESBL genes and harbouring ESBL genes: (4) Screening and confirmatory tests methods. The above information was extracted from each eligible article included and recorded immediately in the data extraction form. The process of the extraction was carried out by four (4) independent reviewers and crosschecked by a fifth reviewer.

### Study quality assessment

After article evaluation for the inclusion and exclusion criteria, all articles included were subjected to a quality assessment using the Joanna Briggs Institute critical appraisal checklist for studies reporting prevalence data [11]. The appraisal tool has 9 questions that were answered either; Yes (Y), No (N), Unclear (UC), or Not applicable (NA). Scores were awarded as; Y = 1, N = 0, UC = 0, and NA attracted no score. Based on the scores the quality of the studies was graded; studies with ≤ 50% scores were deemed low-quality studies. Those with > 50%—69% were termed moderate quality studies. While high-quality studies were those with ≥ 70% scores. The critical appraisal was carried out by four independent reviewers and cross-checked by two other reviewers.

### Meta-analysis

#### Statistical assessment

MetaXL software (add-in for Microsoft Excel) was used for the quantitative analysis of the extracted data. The meta-analysis and pooling of the prevalence estimate (with the 95% confidence interval) were done using the quality effect (QE) model by employing (the transformed) double arcsine method.

#### Assessment of heterogeneity

Estimation of statistical heterogeneity amongst the included studies was done using the *X*.^*2*^

Test, Cochrane Q, and *I*^*2*^ statistics. An *I*^*2*^ value of 0 to ≤ 40% was considered low heterogeneity, > 40% to 60% was regarded as moderate heterogeneity, > 60% to 75% was considered substantial heterogeneity, and > 75% to 100% was considered high heterogeneity.

#### Sensitivity analysis

Sensitivity analysis was done based on leave-one-out model to identify the studies that greatly influence the result of the meta-analysis.

#### Subgroup analysis and meta-regression

Subgroup analysis and meta-regression were conducted to identify the moderators of heterogeneity in the included studies. The factors used in the subgroup analysis are country (location) of study, ASEAN country subdivision, year of study publication, study sample size, detection method (ESBL screening and confirmatory tests) used, study quality and weight of the study in the meta-analysis. In the case of meta-regression, only five factors were used in the univariate analysis. Factors with significant univariate analysis were all used in the multivariate analysis. Due to the low statistical power of the meta-regression 0.25 was considered the significant *p*-value.

#### Publication bias

A funnel plot was constructed to examine for publication bias an asymmetry was observed on the funnel plot. Thus, an additional assessment using the Doi plot to estimate the symmetry of the funnel plot was carried out. Subsequently, Egger’s test was conducted to test the significance of the asymmetry.

## Results

### Study selection process and characteristics of included studies

**Fig. 1:**
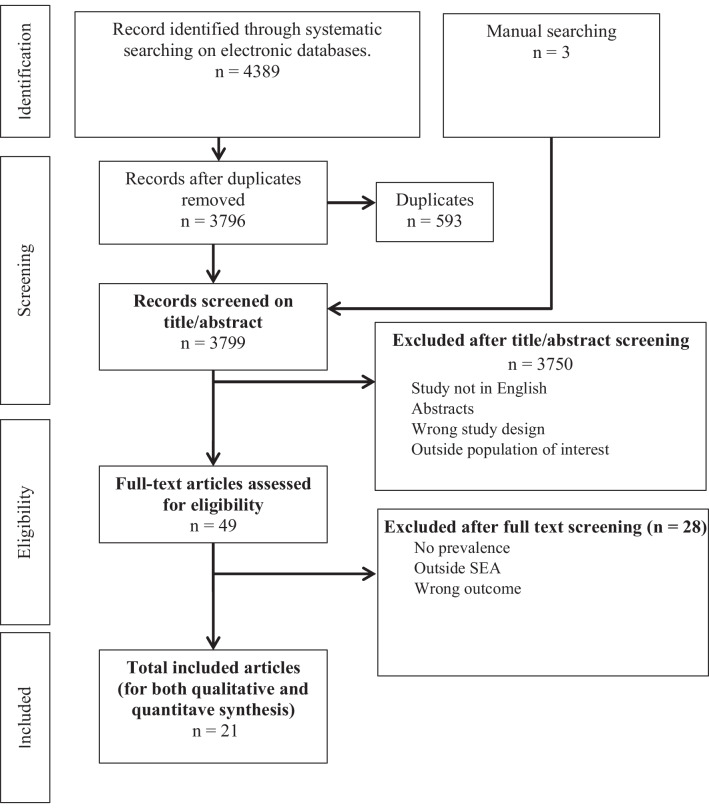
PRISMA Flow diagram

After the completion of database searches, a total of 4389 citations were obtained. In addition, three studies were identified from the manual references search and the searches conducted on internet search engines. Of the total citations, 593 duplicates were removed and 3799 articles were screened on title/abstract. After title/abstract screening, 49 articles were subjected to full-text screening (Additional file 1: Table S1). Finally, 21 articles (Additional file 1: S5 File) were included in this SR&MA (Fig. 1).

The 21 included studies were conducted in eight countries out of the 11 countries in SEA. Malaysia has the highest number of studies [3, 16–18, 20, 22] constituting 28.6% of the total studies included in this SR&MA. Five studies [4, 13–15, 27] from Thailand, four from Indonesia [29–31, 38], two from Vietnam [8, 36] and one each from Singapore [39], Cambodia [37], Philippines [35], and Myanmar [5] were included in the review (Table 1). Of the 21 included studies seven reported MDR prevalence of *K. pneumoniae* while 20 studies reported ESBL prevalence (Table 1). The total sample size of all included studies is 3706 (ranging from 17 to 1001) among varied sampled populations (Table 1). Respiratory infection constitutes the major site of infection and 19% of the samples used in the included studies are archived samples (Table 2). The majority of the included studies used multiple sample sources with a blood sample being the most frequently used (Table 2).

**Table 1:** Characteristics of included studies

Author	Country of study	Period of sampling	Year of publication	Study design	Sample size	Prevalence assessed	
						MDR	ESBL
[20]	Malaysia	September-December 2014	2020	–	97	Y	Y
[13]	Thailand	December 2004–May 2005	2008	–	1001	N	Y
[8]	Vietnam	February–September 2015	2019	Prevalence Survey	57	Y	Y
[3]	Malaysia	2010–2012	2015	Investigation/Survey	93	N	Y
[39]	Singapore	1st May 2014–30th April 2016	2019	-	140	N	Y
[38]	Indonesia	2015	2021	Investigation/Survey	94	N	Y
[37]	Cambodia	2012	2019	Prevalence study	196	N	Y
[27]	Thailand	April 2004-August 2005	2008	Investigation/Survey	31	N	Y
[29]	Indonesia	April until October 2013 & April until August 2014	2020	Prospective observational	200	Y	N
[4]	Thailand	January 1 through 30, 2006	2008	–	71	N	Y
[36]	Vietnam	March and June 2010	2013	–	72	N	Y
[16]	Malaysia	1st June–31st August 2017	2021	Prospective cohort	139	Y	Y
[5]	Myanmar	January 2018	2021	Prevalence	191	N	Y
[35]	Philippines	August–November 2017	2018	Prevalence	32	Y	Y
[15]	Thailand	August 2000-January 2001	2004	Prospective study	400	N	Y
[22]	Malaysia	2009 and 2012	2016	Cross-sectional Descriptive study	141	N	Y
[31]	Indonesia	–	2019	Prospective cohort	72	N	Y
[17]	Malaysia	2004	2009	–	51	Y	Y
[30]	Indonesia	January–April 2005	2010	Investigation/Survey	291	N	Y
[14]	Thailand	July 1, 2004, through June 30, 2005	2007	–	320	N	Y
[18]	Malaysia	2013	2017	Investigation/Survey	17	Y	Y

**Table2:** Sampling characteristics of the included studies

Study	Studied population	Site(s) of infection	Sample type	Source of sample			
				Blood	Urine	Sputum	Others (specify)
[20]	–	–	Archived isolates	Y	Y	Y	Bronchoscopic aspirates, wound tissue, swab, pus, poc, fluid, slough and bone
[13]	patients with HA Infections	–	Fresh clinical specimens	Y	Y	Y	Pus/exudate
[8]	Children	Respiratory, gastrointestinal, cardiovascular & blood	Fresh clinical specimens	Y	N	N	Tracheal fluid, Nasopharynx
[3]	Patients attending University of Malaya medical centre	–	Archived isolates	–	–	–	–
[39]	Patients with KP visceral organ abscesses	Liver, intestinal, urinary tract	Fresh clinical specimens	Y	N	N	Abscess aspirate
[38]	UTI Patients	Urinary tract	clinical isolates	–	–	–	–
[37]	Children/Adolescents	Intestinal	Fresh clinical specimens	N	N	N	Faecal samples
[27]	–	–	Clinical isolates	Y	N	N	–
[29]	ICU patients/HCWs	Rectal & throat	Fresh clinical specimens	Y	Y	N	Lower respiratory tract, tissue & wound
[4]	Adult patients with HCAI	Urinary tract & bloodstream	Archived isolates	–	–	–	–
[36]	–	–	Clinical isolates	–	–	–	–
[16]	Preterm infants	Respiratory	Fresh clinical specimens	N	N	N	Tracheal secretions, stool
[5]	Patients with respiratory infections	Respiratory	Clinical isolates	N	N	Y	–
[35]	In-and out-patients attending teaching hospital in the Philippines	–	Clinical isolates	Y	Y	Y	Wound
[15]	Patients attending Siriraj Hospital, Mahidol University Bangkok	–	Clinical isolates	Y	Y	Y	–
[22]	Inpatients attending Hospital Parkar Sultannah Fatimah	–	Clinical isolates	–	–	–	–
[31]	Patients who suffered from bloodstream infection	Bloodstream	Fresh clinical specimens	Y	N	N	–
[17]	Patients attending five different hospitals located in peninsula Malaysia	–	Fresh clinical specimens	Y	Y	Y	Tracheal aspirates, catheter tips, pus and swab samples
[30]	–	–	Fresh clinical specimens	Y	Y	Y	Wound, Stool and CSF specimens
[14]	Adults with ESBL-EC or ESBL-KP infection	–	Fresh clinical specimens	Y	Y	N	Ascitic fluid, Tracheal aspirate
[18]	–	–	Archived isolates	Y	Y	Y	Tissue, Swab, Drainage fluid and Tracheal secretions

**Table 3:** Summary of the subgroup analysis result

Subgroups	Number of studies	Pooled prevalence		Heterogeneit*y*	
		%	95% CI	*I*^*2*^	*P*
Year of publication	20				
2004–2009	6	14	1–32	97	< 0.0001
2010–2015	3	45	0–100	99	< 0.0001
2016–2021	11	54	22–85	98	< 0.0001
Weight-based	20				
> 10	2	9	0–25	98	< 0.0001
4–9	4	23	12–35	94	< 0.0001
< 4	14	70	44–93	98	< 0.0001
Sample size	20				
< 50	8	54	31–77	90	< 0.0001
> 50–< 100	5	75	22–100	99	< 0.0001
> 100–< 150	2	40	0–100	100	< 0.0001
> 150–< 400	3	13	0–39	99	< 0.0001
> 400	2	16	0–31	97	< 0.0001
Study quality	20				
Low	4	51	11–90	91	< 0.0001
Moderate	9	58	18–96	99	< 0.0001
High	7	13	0–27	96	< 0.0001
					< 0.0001
ASEAN Country (subdivision)	20				
1st ASEAN	16	26	0–56	99	< 0.0001
Other ASEAN	4	55	0–100	98	< 0.0001
Specific country	20				
Malaysia	6	76	39–100	98	< 0.0001
Thailand	5	13	0–26	97	< 0.0001
Vietnam	2	98	0–100	96	< 0.0001
Singapore	1	6	0–10	–	–
Indonesia	3	46	0–100	99	< 0.0001
Cambodia	1	51	0–42	–	–
Myanmar	1	35	0–28	–	–
Philippines	1	7	0–57	–	–
Screening and confirmatory test	20				
Disk diffusion	3	7	0–39	97	< 0.0001
Disk combination	6	23	0–52	99	< 0.0001
E-test	2	1	98–100	0	< 0.0001
PCR	3	67	0–100	99	< 0.0001
DDST	4	31	0–90	94	< 0.0001
Micro-dilution	1	57	42–71	–	–
Not specific	1	6	0–10	–	–

### Risk of bias (quality) assessment

The quality of the included studies was assessed using the JBI appraisal tool for prevalence studies. Four of the 21 included studies are of low quality, 11 of moderate quality and the remaining are of high quality (Additional file 1: Table S2).

### Outcomes

#### Primary outcome

**Fig. 2:**
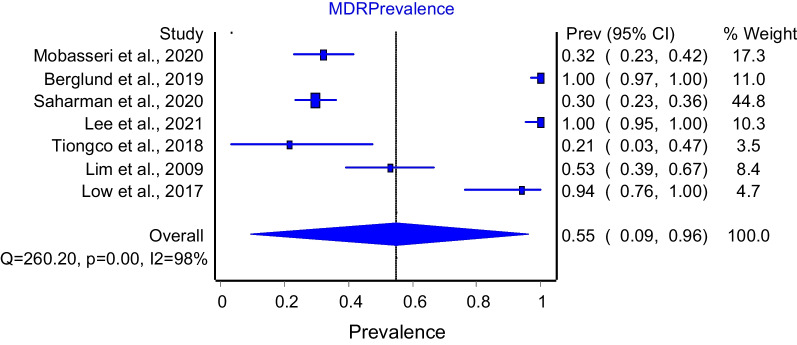
Forest plot of overall meta-analysis of *K. pneumoniae* MDR prevalence in SEA

The prevalence of the seven studies that reported the prevalence of MDR associated with *K. pneumoniae* was pooled for the meta-analysis. The overall MDR prevalence obtained from the seven studies is 55% (95% confidence interval [CI] 9–96). Cochrane Q value (Q; 260.2), *I*^*2*^; 98%, and *p* < 0.0001 (Fig. 2).

Since the number of studies that assessed the MDR is not up to ten publication bias, subgroup analysis and sensitivity analysis were not conducted for the primary outcome.

#### Secondary outcomes

**Fig. 3:**
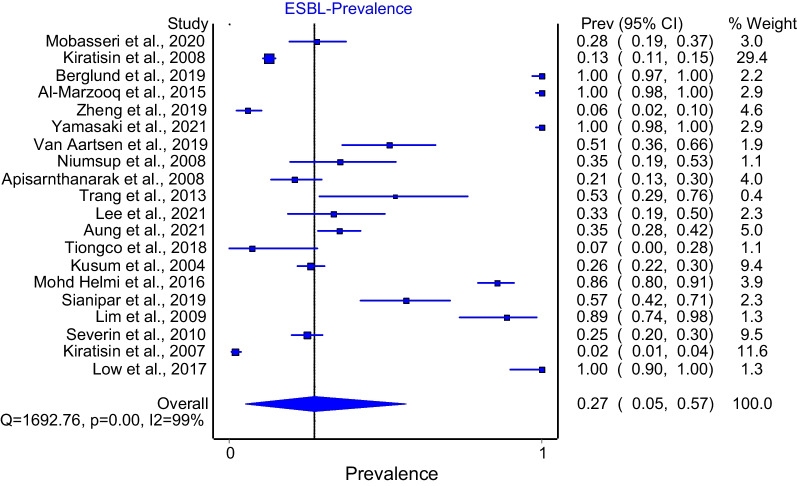
Forest plot of overall meta-analysis of *K. pneumoniae* ESBL prevalence in SEA

ESBL prevalence: Twenty studies were included in the meta-analysis to obtain the overall prevalence estimate of ESBL *K. pneumoniae*. The pooled prevalence obtained for the ESBL of *K. pneumoniae* in SEA is 27% (CI 5–57, Q: 1692.76, *I*^2^: 99% *p* < 0.0001). The meta-analysis result is summarized in the Additional file 1: Table S3 and Fig. 3 gives the graphical presentation of the result.

### Sensitivity analysis

**Fig. 4:**
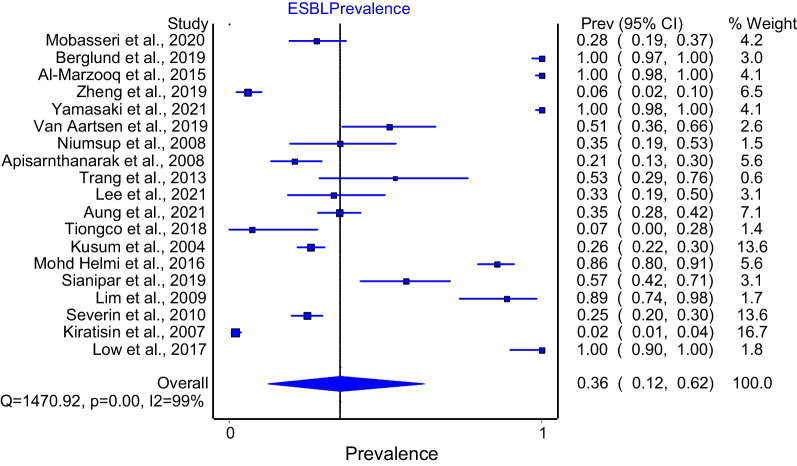
Forest plot of sensitivity analysis of *K. pneumoniae* ESBL prevalence in SEA

The study [13] with the highest weight (and largest sample size) was removed for the sensitivity analysis. The excluded study had a significant impact on the overall estimate giving a prevalence of 36% (Fig. 4).

### Subgroup analysis and meta-regression

To explore the factors responsible for the observed heterogeneity subgroup and meta-regression analyses were conducted. Seven pre-specified factors were used for the subgroup analysis (Table 3). Graphical presentations of the different subgroup analyses are presented in the Additional file 1: Figures S1.

**Table 4:** A meta-regression analysis

Univariate meta-regression			Multivariate meta-regression	
Predictors (factors)	*R*^*2*^ (%)	*P* value	*R*^2^ (%)	*P* value
Sample size	15.9	0.082	93.5	0.102
Year of publication	9.3	0.192		
Study quality	12.1	0.133		
Test type	54.3	0.072		
Country of study	57.1	0.100		

*R*^2^: The proportion of the effect of covariates on heterogeneity (between-study variance)

For the meta-regression, 5 factors were used in the univariate analysis. The effect proportions (*R*^2^) of the covariates on heterogeneity and their corresponding *p* values are summarized in Table 4. Also, the overall proportion effect of all the factors revealed by the multivariate analysis was 93.5% (Table 4).

### Publication bias

**Fig. 5:**
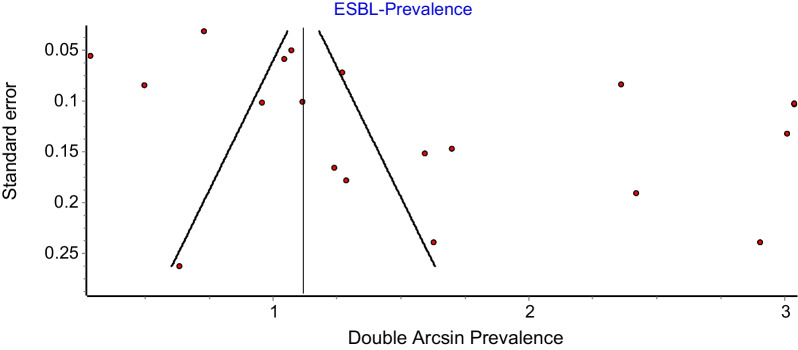
Funnel plot of meta-analysis of *K. pneumoniae* ESBL prevalence in SEA

A funnel plot was constructed to examine for publication bias (Fig. 5).

**Fig. 6:**
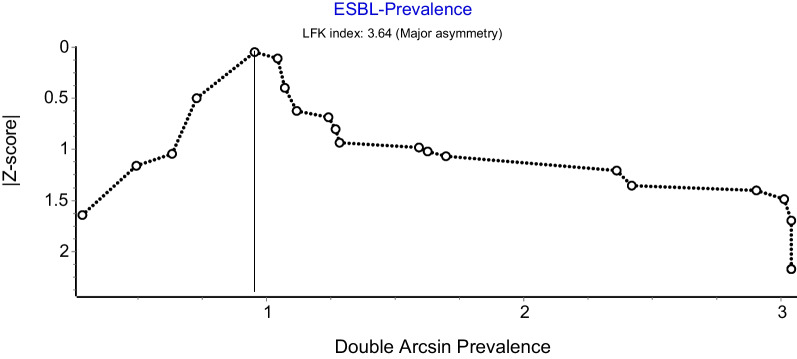
Doi plot of meta-analysis of *K. pneumoniae* ESBL prevalence in SEA

There was an observed asymmetry on the funnel plot and therefore a further assessment using the Doi plot to evaluate the symmetry of the funnel plot was carried out (Fig. 6). The Doi plot showed a major asymmetry with an LFK index of 3.64. Also, Egger’s regression test was conducted to test the significance of the asymmetry and the *p*-value is 0.74.

Predominant ESBL occurring genes: ESBL genes were not determined in four of the included studies [15, 31, 35, 39]. The remaining 17 studies determined different ESBL genes totalling 870 of which *bla*_CTX-M_ is the most frequently identified in 10 (58.8%) studies (Table 5). *bla*_CTX-M_ occurred 463 times (53.2%) out of the 870 identified genes. Other identified ESBL genes include *bla*_SHV_ (29.4% of studies), *bla*_TEM,_ (5.9%), and *bla*_OXA_ (5.9%).

**Table 5:** Characteristics of determined ESBL genes and the detection methods

Study	Predominant ESBL gene		Harbouring ESBL genes		Identification methods	
	Name	Frequency (n)	Number	Specific genes	Screening	Confirmatory
[20]	*bla*_TEM_	26 (27)	27	CTX-M, TEM, SHV	E-test	Disk diffusion
[13]	*bla*_CTX-M_	127(127)	125	CTX-M, TEM, SHV,OXA,VEB	E-test	Combination disk
[8]	*bla*_OXA_	52(57)	–	OXA, TEM, SHV	–	E-test
[3]	*bla*_CTX-M_	85(93)	20	CTX-M, SHV, OXA, TEM	Disk diffusion	E- test
[39]	–	–	–	–	–	–
[38]	*bla*_CTX-M_	84(94)	90	CTX-M, TEM, SHV	Disk diffusion	PCR amplification
[37]	*bla*_CTX-M_	14(22)	–	CTX-M, SHV	–	Disk combination
[27]	*bla*_SHV_	26(31)	22	SHV, TEM, CTX-M	DDST	Combination disk
[29]	–	–	–	–	–	–
[4]	*bla*_SHV_ & *bla*_*CTX-M*_	18(20)	13	CTX-M, SHV, VEB	E-test	DDST
[36]	*bla*_*CTX-M*_	9(9)	6	CTX-M, TEM	E-test	DDST
[16]	*bla*_SHV_	33(36)	32	SHV, TEM, CTX-M, OXA	–	Disk diffusion
[5]	*bla*_CTX-M_	63(67)	3	CTX-M, TEM, SHV	–	Multiplex PCR
[35]	–	–	–	–	–	DDST
[15]	–	–	–	–	Disk diffusion, E-test	Disk combination
[22]	*bla*_*SHV*_	106(121)	35	SHV, TEM, CTX-M	Disk diffusion	Disk combination
[31]	–	–	–	–	–	Micro-dilution broth
[17]	*bla*_*SHV*_	46(51)	4	SHV, CTX-M, OXA, TEM	Disk diffusion	DDST
JA et al., 2010	*bla*_CTX-M_	40(72)	32	CTX-M, SHV	Disk diffusion	Disk combination
[14]	*bla*_CTX-M_	6(6)	6	CTX-M, SHV, TEM	E-test	Disk diffusion
[18]	*bla*_CTX-M_	17(17)	16	TEM, SHV, OXA, CTX-M	–	PCR

ESBL harbouring genes: The total number of harboured genes (i.e., more than one gene occurring in an isolate) in all the studies is 431 (Table 5). The commonest harboured gene combinations were *bla*_CTX-M-_*bla*_TEM-_* bla*_*SHV*_ (6 out of 16 studies) followed by *bla*_CTX-M-_*bla*_TEM-_* bla*_*SHV-*_*bla*_OXA_ (3 out of 16).

Site of infection for MDR and ESBL *K. pneumoniae*: The commonest site of infection identified in the included studies is the respiratory tract. Others include the urinary tract, intestinal tract, liver, throat, bloodstream, and cardiovascular (Table 2).

Screening and confirmatory method: Five out of 21 studies, used E–test, six used a disk diffusion test, and one used DDST as screening tests for ESBL. While the remaining nine studies did not specify the test type used (Table 2). The commonly used confirmatory test is the disk combination test (6/21). Followed DDST (4/21), PCR (3/21), disk diffusion (3/21), E-test (2/21), and micro-dilution (1/21). While two studies did not specify the method used for confirmation (Table 2).

Sex distribution of ESBL prevalence: This outcome was not assessed in any of the included studies.

## Discussion

Our primary outcome which is the overall MDR KP prevalence was achieved using the seven studies [8, 16–18, 20, 29, 35] that reported MDR KP prevalence. The meta-analysis of these seven studies demonstrated an overall MDR KP prevalence of 55% (CI 9–96). The pooled prevalence of MDR KP obtained in this study is similar to the 32.8% reported in a systematic review that evaluated the global prevalence of nosocomial MDR KP [21]. Although slightly higher than the global average, the result obtained in this study is still comparable to the 72.4%, 39.6%, and 35.4% prevalence reported for South America, Asia, and the Middle East regions respectively in the subgroup analysis of the same study [21]. The study [21] also, reported a similar prevalence of 72%, 64.3%, and 55% for the Czech Republic, Saudi Arabia, and Iran respectively at individual country subgroup analysis. The result of this analysis has further confirmed the hyperendemicity of MDR KP in the SEA subregion. All the seven studies pooled in this meta-analysis are from developing countries of SEA. Expectedly as compared to other developing countries, the MDR prevalence is high. Other possible reasons for the high prevalence include the high transmission rate of nosocomial MDR KP in the subregion and prolonged hospitalization.

Our study also analysed the prevalence of ESBL KP in the subregion. The overall prevalence estimate of ESBL KP is 27% (CI 5–57). An equally high ESBL (Enterobacteriaceae) prevalence of 42% was reported in a similar review in the East Africa subregion [32]. In another study, the global prevalence of ESBL Enterobacteriaceae was reported to be 25% [19]. Similarly, another systematic review reported ESBL KP prevalence in Africa ranging from 0.7% to as high as 75.8% [33]. However, the ESBL prevalence found in our analysis is higher than those obtained in Europe (5%), South America (4%), and North America (3%) [19]. Our study has further confirmed that ESBL KP prevalence is high in the SEA subregion comparable to the global average. It might not also be out of place to assume that the developing regions contribute more to the global prevalence of ESBL KP. Because studies have reported low prevalence from the developed regions. Reasons for high MDR KP could as well explain for the reported high prevalence of ESBL KP.

To ensure reliability, a quality effect model was used for the meta-analysis. Expectedly, however, there was high heterogeneity between included studies for the ESBL KP prevalence. Thus, the predesigned subgroup and meta-regression analyses with factors anticipated to moderate the heterogeneity were conducted. The effect of the factors on the ESBL KP prevalence was evaluated individually (in the subgroup and univariate analysis) and collectively in the multivariate analysis. Study location subgroup analysis was conducted at two levels; individual country and ASEAN countries subdivision levels. The country-level subgroup analysis revealed significant differences in the country's prevalence. The different ESBL KP prevalence is 76%, 13%, 98%, 6%, 46%, 51%, 35%, and 7% for Malaysia, Thailand, Vietnam, Singapore, Indonesia, Cambodia, Myanmar, and the Philippines respectively. The obtained prevalence for each of the countries is high except for the Philippines and Singapore. The high prevalence for the different countries in this study is comparable to what has been reported for other developing countries in some studies. A prevalence of 43.5% was reported for ESBL KP in Iran [7]. Many other developing countries have also reported high ESBL KP prevalence ranging from 38 to 55% [2, 7]. On the other hand, and expectedly so, the prevalence in Singapore was low. Likely because Singapore is a developed country. However, the low prevalence seen in the Philippines might be due to the very small sample size of the Philippines study. Similarly, the ASEAN country subdivision analysis shows that the first ASEAN countries have a lower prevalence than the other ASEAN countries. The country-level results from the analysis of this study have shown that ESBL KP prevalence in the subregion varies from country to country. It is also obvious that developing countries have high ESBL KP prevalence which might be due to some common factors among the countries. These factors may include a proportion of severely ill patients, prolonged hospitalization, and antibiotic policy among others [12].

In the year of publication subgroup analysis, the prevalence showed an increasing pattern. The result revealed an ESBL KP prevalence increase from 14% in the period 2004–2009 to as high as 54% between 2016 and 2021. The progressive increasing prevalence of ESBL KP revealed in this review, implies that inhabitants of the subregion are highly at risk of infections due to ESBL KP. The reasons for the liberal increase of ESBL KP prevalence in the study region might be a result of irrational antibiotic use, records of prolonged illnesses and hospitalization in most developing countries. Therefore, frequent research on the subject matter needs to be undertaken to closely monitor the ESBL KP prevalence subsequently in the subregion. Additionally, regular surveillance would help in further prevention of ESBL-KP prevalence in the study region. Our review also did a subgroup analysis on the types of tests (screening and confirmatory) used in the detection of ESBL. To determine how the methods moderate ESBL prevalence. There were six methods used for the detection of ESBL in the studies included (disk diffusion, disk combination, E-test, DDST, micro-dilution and PCR). However, some studies did not specify the test used. It is also a known fact that the use of different detection methods produces high heterogeneity in prevalence study meta-analysis [10]. The commonest used detection method is the disk combination which has a pooled prevalence of 23%. The prevalence of the disk combination method is however lower than the prevalence of DDST and PCR with 31% and 67% respectively. Likely due to the high sensitivity associated with PCR and DDST which needs to be promoted and adopted for the detection of ESBL. To further explore the factors contributing to heterogeneity in our meta-analysis, we did sample size, study quality and study-weight subgroup analysis. Regarding sample size analysis, studies with a small sample size tend to have higher prevalence when compared to larger sample size studies. Equally, larger weighted studies showed higher prevalence than the small weight studies. In addition, because there are multiple possible sources of heterogeneity a meta-regression (univariate and multivariate) was done using the top five most likely factors. Of the five factors examined using univariate analysis, four (year of publication, study quality, methods used, and study location) explained the existing heterogeneity by 9.3%, 12.1%, 54.3%, and 57.1% respectively. While the four covariates collectively accounted for 93.5% of the heterogeneity significantly in a multivariate analysis. This, therefore, implies that these factors play a substantial role in the variation observed from reported ESBL KP prevalence in the subregion.

Globally, concerns are rising about the effect ESBL producing bacteria in the development of treatments against bacterial infection. Thus, our review sought to evaluate the major ESBL occurring genes from KP isolates in the subregion. The identified ESBL genes in this study were in line with the three known major genes; TEM, SHV, and CTX-M types [9]. However, in this review CTX—M was the predominantly identified ESBL gene type. The known pattern of spread is that CTX—M occurs predominantly in *E. coli* and mostly in community-acquired infections [9]. Our result is equally in agreement with the rising prevalence of ESBL producing bacteria in Asia [9]. Although the review focused on KP in clinical settings which may imply that CTX-M can likewise be a predominant gene type in KP. Equally, the two commonest ESBL gene co-harbouring detected in our review are the CTX—M-TEM-SHV and CTX—M-TEM-SHV-OXA types. Implying the increased resistance warranting more continued robust antimicrobial resistance surveillance. Additionally, from the result of this review, it is not out of place to conclude that we are dealing with an allodemic situation regarding ESBL KP in SEA. This situation therefore, will require an environmental control rather than the classical approach [6].

Moreover, aside from assessing for the MDR and ESBL prevalence, and ESBL gene types, we examined the sites of ESBL KP infection and the screening and confirmatory test used in ESBL detection. Respiratory and urinary tracts were the two commonest identified infection sites for ESBL KP in this review. This information is relevant in the identification of an at-risk population for ESBL KP infection. Knowing this will also assist in the development and implementation of prevention and control interventions at different levels of healthcare management. Information about detection methods is equally vital for the success of infection prevention and control measures. As it will guide on the choice of the most effective technique available. Thus, improving prompt diagnosis which will in turn enhance good clinical outcomes. Therefore, the information on the most commonly used test types provided in this report is pivotal. The outcome of the sex distribution of ESBL KP prevalence, however, was not assessed because none of the included studies reported the outcome. Thereby providing a very vital research gap that needs to be explored.

It is worthy of note that this is the first SR&MA as far as we know to comprehensively evaluate the epidemiology of MDR and ESBL *K. pneumoniae* (KP) in the SEA subregion. This study we believe is comprehensive because we robustly evaluated six important outcomes associated with the epidemiology of MDR KP in clinical settings; overall MDR KP prevalence, ESBL KP prevalence, predominant ESBL genes in KP isolates, harbouring ESBL KP genes, frequent sites of infection, and commonly used screening and confirmatory tests for ESBL detection. In this review, we also determined the factors contributing to the heterogeneity between the included studies. However, the study is not without limitations because only English language publications were included in the review. Also, the review based on the included studies did not cover all the countries in the subregion. Studies were not identified from three countries in the subregion; Laos, Brunei, and Timor – Leste. This may have an implication in the generalization of the findings. Therefore, the interpretation of the review findings should be made in the context of the limitations.

## Conclusions

This study has shown that MDR and ESBL KP are very common in SEA. However, there are many variables that can affect the prevalence of ESBL KP, including study location, study quality, sample size, detection methodology, and publication year. The study also demonstrates that the sub-region's distribution of the EBL KP gene favors an allodemic scenario. Therefore, offering long-term environmental solutions for the containment of the threat requires significant multilateral collaboration between member countries.

### Supplementary Information

**Additional file 1:** **Figures S1.** Subgroups analysis forest plots. **File S1.** PRISMA 2020 checklist. **File S2.** PRISMA-P 2015 checklist. **File S3.** Study protocol. **File S4.** De-duplicated citations. **File S5.** Included studies. **Table S2.** JBI critical appraisal checklist. **Table S3.** ESBL summary.

## Data Availability

Not applicable.
