# Supplementary material for: Epidemiology of multidrug-resistant Klebsiella pneumoniae infection in clinical setting in South-Eastern Asia: a systematic review and meta-analysis
Source: Antimicrob Resist Infect Control. 2023 Dec 7;12:142. doi: 10.1186/s13756-023-01346-5 (PMC10704709; doi:10.1186/s13756-023-01346-5)
Supplement: Supplementary file 1 — Additional file 1: Figures S1. Subgroups analysis forest plots. File S1. PRISMA 2020 checklist. File S2. PRISMA-P 2015 checklist. File S3. Study protocol. File S4. De-duplicated citations. File S5. Included studies. Table S2. JBI critical appraisal checklist. Table S3. ESBL summary. [file 13756_2023_1346_MOESM1_ESM.zip › Supplementary materials/S1 Figures - Subgroup analysis]

Figure 1: Forest plot for country-level subgroup analysis.

Figure 2. Forest plot for ASEAN country subdivision

Figure 3. Forest plot for the year of publication subgroup analysis
