# Supplementary material for: Epidemiology of multidrug-resistant Klebsiella pneumoniae infection in clinical setting in South-Eastern Asia: a systematic review and meta-analysis
Source: Antimicrob Resist Infect Control. 2023 Dec 7;12:142. doi: 10.1186/s13756-023-01346-5 (PMC10704709; doi:10.1186/s13756-023-01346-5)
Supplement: Supplementary file 1 — Additional file 1: Figures S1. Subgroups analysis forest plots. File S1. PRISMA 2020 checklist. File S2. PRISMA-P 2015 checklist. File S3. Study protocol. File S4. De-duplicated citations. File S5. Included studies. Table S2. JBI critical appraisal checklist. Table S3. ESBL summary. [file 13756_2023_1346_MOESM1_ESM.zip › Supplementary materials/S2 Table JBI critical appraisal checklist]

S2 Table

Joanna Briggs Institute critical appraisal checklist for studies reporting prevalence data

| **S/N** | **Study** | **Q1** | **Q2** | **Q3** | **Q4** | **Q5** | **Q6** | **Q7** | **Q8** | **Q9** | **Total score** | | | | **%** |
| --- | --- | --- | --- | --- | --- | --- | --- | --- | --- | --- | --- | --- | --- | --- | --- |
|  |  |  |  |  |  |  |  |  |  |  | **Y** | **N** | **UC** | **NA** |  |
| 1 | Mobasseri et al., 2020 | NA | NA | N | Y | NA | Y | UC | NA | Y | 3 | 1 | 1 | 4 | 60.0 |
| 2 | Kiratisin et al., 2008 | Y | Y | Y | Y | UC | Y | UC | Y | Y | 7 | 0 | 2 | 0 | 77.8 |
| 3 | Berglund et al., 2019 | Y | UC | N | Y | Y | Y | UC | NA | Y | 5 | 1 | 2 | 1 | 62.5 |
| 4 | Al-Marzooq et al., 2015 | NA | NA | N | N | Y | Y | UC | Y | Y | 4 | 2 | 1 | 2 | 57.1 |
| 5 | Zheng et al., 2019 | Y | Y | Y | UC | Y | UC | Y | Y | UC | 6 | 0 | 3 | 0 | 66.7 |
| 6 | Yamasaki et al., 2021 | N | Y | N | UC | Y | Y | UC | Y | Y | 5 | 2 | 2 | 0 | 55.6 |
| 7 | Van Aartsen et al., 2019 | N | Y | N | Y | Y | Y | UC | Y | UC | 5 | 2 | 2 | 0 | 55.6 |
| 8 | Niumsup et al., 2008 | NA | NA | N | N | Y | Y | UC | NA | Y | 3 | 2 | 1 | 3 | 42.9 |
| 9 | Saharman et al., 2020 | Y | Y | Y | Y | Y | Y | UC | Y | UC | 7 | 0 | 2 | 0 | 77.8 |
| 10 | Apisarnthanarak et al., 2008 | Y | Y | N | Y | Y | Y | UC | Y | Y | 7 | 1 | 1 | 0 | 77.8 |
| 11 | Trang et al., 2013 | N | UC | N | N | Y | Y | UC | NA | Y | 3 | 3 | 2 | 1 | 37.5 |
| 12 | Lee et al., 2021 | Y | Y | N | Y | Y | Y | UC | Y | Y | 7 | 1 | 1 | 0 | 77.8 |
| 13 | Aung et al., 2021 | Y | Y | N | N | Y | Y | UC | NA | Y | 5 | 2 | 1 | 1 | 62.5 |
| 14 | Tiongco et al., 2018 | N | Y | N | N | Y | Y | UC | NA | Y | 4 | 3 | 1 | 1 | 50.0 |
| 15 | Kusum et al., 2004 | N | Y | Y | N | Y | Y | UC | NA | Y | 5 | 2 | 1 | 1 | 62.5 |
| 16 | Mohd Helmi et al., 2016 | N | Y | N | N | Y | Y | UC | Y | Y | 5 | 3 | 1 | 0 | 55.6 |
| 17 | Sianipar et al., 2019 | Y | Y | N | UC | Y | Y | UC | Y | Y | 6 | 1 | 2 | 0 | 66.7 |
| 18 | Lim et al., 2009 | N | Y | N | N | Y | Y | UC | NA | Y | 4 | 3 | 1 | 1 | 50.0 |
| 19 | Severin et al., 2010 | Y | Y | Y | N | Y | Y | UC | NA | Y | 6 | 1 | 1 | 1 | 75.0 |
| 20 | Kiratisin et al., 2007 | Y | Y | Y | Y | Y | Y | UC | NA | Y | 7 | 0 | 1 | 1 | 87.5 |
| 21 | Low et al., 2017 | NA | NA | N | N | Y | Y | UC | Y | Y | 4 | 2 | 1 | 2 | 57.1 |
