# Supplementary material for: Epidemiology of multidrug-resistant Klebsiella pneumoniae infection in clinical setting in South-Eastern Asia: a systematic review and meta-analysis
Source: Antimicrob Resist Infect Control. 2023 Dec 7;12:142. doi: 10.1186/s13756-023-01346-5 (PMC10704709; doi:10.1186/s13756-023-01346-5)
Supplement: Supplementary file 1 — Additional file 1: Figures S1. Subgroups analysis forest plots. File S1. PRISMA 2020 checklist. File S2. PRISMA-P 2015 checklist. File S3. Study protocol. File S4. De-duplicated citations. File S5. Included studies. Table S2. JBI critical appraisal checklist. Table S3. ESBL summary. [file 13756_2023_1346_MOESM1_ESM.zip › Supplementary materials/S3 File MDR Klebsiella pneumoniae Protocol]

S1 File. Study protocol

**Title: Protocol for Epidemiology of Multidrug Resistant *Klebsiella pneumoniae* Infection in Clinical Setting in South-Eastern Asia: A Systematic Review and Meta-analysis**

**Registration:**Registered

Authors: Salawudeen Adamu^1,2^, Yakubu Egigogo Raji^6^, Garba Gidandawa Jibo^1,3^, Mohd Nasir Mohd Desa^4^, Hui Min Neoh^5^, Siti Norbaya Masri^1^ and Tengku Zetty Maztura Tengku Jamaluddin^1,^*

**^1^** Department of Medical Microbiology, Faculty of Medicine and Health Sciences, Universiti Putra Malaysia 1; [asalawudeen@gsu.edu.ng](mailto:asalawudeen@gsu.edu.ng) (S.A); satikanya@gmail.com (G.G.J); [sitinorbaya@upm.edu.my](mailto:sitinorbaya@upm.edu.my) (S.N.M) [tengkuzetty@upm.edu.my](mailto:tengkuzetty@upm.edu.my) (TZMTJ)

**^2^** Department of Microbiology, Faculty of Science, Gombe State University, Tudun Wada, Gombe, Gombe State, Nigeria: [asalawudeen@gsu.edu.ng](mailto:asalawudeen@gsu.edu.ng) (S.A)

**^3^** Department of Microbiology, Faculty of Life Sciences, Kebbi State University of Science and Technology, Aliero, Nigeria; [satikanya@gmail.com](mailto:satikanya@gmail.com) (G.G.J)

**^4^** Department of Biomedical Sciences, Faculty of Medicine and Health Sciences, Universiti Putra Malaysia: [mnasir@upm.edu.my](mailto:mnasir@upm.edu.my) (M.N.M.D)

**^5^** UKM Medical Molecular Biology Institute (UMBI), UKM Medical Centre, Jalan Ya’acob Latiff, Bandar Tun Razak, 56000 Kuala Lumpur, Malaysia: [hui-min@ppukm.ukm.edu.my](mailto:hui-min@ppukm.ukm.edu.my) (H.M.N)

**^6^** Department of Pathology, Clinical Microbiology Unit, College of Health Sciences, Ibrahim Badamasi Babangida University Lapai, Nigeria, [rajiegigogoy@ibbu.edu.ng](mailto:rajiegigogoy@ibbu.edu.ng) (YR)

***** Correspondence: tengkuzetty@upm.edu.my; Tel.: +60162035743

**Authors’ contributions:** Conception of research idea (TZMTJ), Literature review (SA), Research protocol design (SA), Study appraisal (TZMTJ, MNMD and HMN), Data extraction (SA, GGJ, SNM, YR and TZMTJ), Data analysis and interpretation of results (TZMTJ, SNM, HMN, YR and SA), Manuscript drafting (SA), and review of initial and final draft of manuscript (TZMTJ, MNMD, HMN and SNM)

**Source of Support:** Universiti Putra Malaysia

**Sponsor:** Universiti Putra Malaysia

**Role of sponsor:** The sponsor did not play any role in the development of the study protocol.

**Introduction**

There are concerned issues of global health today and one of them is the rapid spread of

multidrug resistant (MDR) bacteria. (Tanwar et al., 2014) identified high mortality, prolong

illness, a threat to therapeutic, decreased effectiveness of drugs, easy target for

immunocompromised conditions, and high medical cost as the problems associated with

multidrug-resistant bacteria. *Klebsiella pneumoniae* in particular exhibits the quality of

multidrug resistance which enables them to resist last-line antimicrobial medicines such as

colistin, tigecycline, and carbapenems increasingly. This particular bacterium has the ability to

colonize different human systems such as the gastrointestinal tract, nasopharynx as well as to

skin and cause both hospital and community infections (Nguyen et al., 2021).

*Klebsiella pneumoniae* have been prioritized as one of the top three pathogens of international

concerned in 2017 by World Health Organization being clinically important pathogen causing

various infections such as urinary tract infections, respiratory infection, bacteraemia,

pneumonia etc (Yamasaki et al., 2021). MDR *Klebsiella pneumoniae* involves both extended

spectrum beta lactamases (ESBLs) and cabapenemases.

ESBLs are defined as plasmid-mediated and chromosomal enzymes that hydrolyse or

inactivate beta-lactam antibiotics or in other words are enzymes which hydrolyse a wide variety

of beta-lactam antibiotics including oxymino-cephalosporins and aztreonam but inhibited by

beta-lactam inhibitors like clavulanic acid, tazobactam and sulbactam (Abla et al., 2016). ESBL

*Klebsiella pneumoniae* are a serious public health concern globally. ESBL-producing K.

pneumoniae strains have been associated with severe outbreak situations and nosocomial

infections worldwide.

The World Health Organization has estimated the loss of about ten million lives annually and economic output worthy of one hundred trillion USD in danger due to increase in diseases caused by drug resistant organisms (Morrison & Zembower, 2020).

*K. pneumoniae* are understand to be a growing threat in the SEA and very scanty studies are available to addressed the aforementioned challenges caused by the organisms (Aung et al., 2021; Naeemmudeen et al., 2021; Yamasaki et al., 2021). Therefore, there is need for a systematic review and meta-analysis to evaluate the epidemiology of MDR in *K. pneumoniae* in order to generate an adequate data that can filled the gap in the study region.

**Review Questions**

1. What is the overall prevalence of multidrug resistant(MDR) *Klebsiella pneumoniae* in South-Eastern Asia?
2. What is the occurrence of extended spectrum beta lactamases (ESBLs) in MDR *Klebsiella pneumoniae* in South-Eastern Asian Countries?
3. What is the predominant occurrence of ESBL genes in MDR extended spectrum beta lactamase *Klebsiella pneumoniae* in South-Eastern Asia?
4. What is the rate of ESBL gene harbouring in ESBL *Klebsiella pneumoniae* in South-Eastern Asia?
5. What is the incidence of sex ESBL *Klebsiella pneumoniae* infection in hospitals of South-Eastern Asian Countries?
6. What part of the human systems are affected by MDR extended spectrum beta lactamase *Klebsiella pneumoniae*?
7. What is the screening and confirmatory test methods adopted in the isolation of MDR extended spectrum beta lactamase *Klebsiella pneumoniae*?

**Objectives:**

Aim: is to evaluate the epidemiology of multidrug resistant *Klebsiella pneumoniae* infection in clinical setting in South-Eastern Asia by reviewing and summarising relevant literature. In this study, overall MDR *Klebsiella pneumoniae* which include both ESBLs and cabapenemases would be considered

Specific objectives include:

1. To assess the overall proportion (prevalence or incidence) of MDR Klebsiella pneumoniae infection among patients in hospitals of South-Eastern Asia
2. To assess the ESBL *Klebsiella pneumoniae* prevalence among patients in hospitals.
3. To determine the predominant ESBL occurring genes.
4. To examine the rate of isolates harbouring ESBL genes.
5. To assess the sex distribution of ESBL *K. pneumoniae* infection prevalence among the patients in hospitals
6. To assess the site of infection of ESBL *K. pneumoniae*
7. To determine the screening and confirmatory tests methods adopted in ESBL *K. pneumoniae* isolation

**Methods**

This systematic review will be conducted in accordance with the Preferred Reporting Items for Systematic Reviews and Meta-Analysis (PRISMA) guidelines (Moher et al., 2016).

- Inclusion criteria
- Study type: all observational studies (cross sectional, cohort, case control, prevalence surveys) that studied cases of MDR in *K. pneumoniae.*
- Studies conducted in humans among hospital patients will be included.
- Study location: studies conducted in SEA countries will be included.
- Time period: there will be no time limitation placed on the period of publication.
- Age and sex: no restriction
- Language of publication: only studies published in English language will be included.
- Publication type: both peer-reviewed and preprint articles will be included,
- Exclusion criteria
- Studies involving healthcare workers (occupational or work-related) infections will be excluded.
- Studies of community acquired infections or conducted outside clinical/hospital settings will be excluded.
- Studies of MDR in animals will be excluded.
- Studies conducted in countries outside SEA will be excluded.
- Studies of MDR conducted in other bacteria will be excluded.
- Studies of a drug (single drug) resistance in *Klebsiella pneumoniae* will be excluded.
- *In silico, In vitro,* as well as *In vivo* (using animal models) studies will be excluded.
- Studies with incomplete data will be excluded.
- Letters, books, book chapters, dissertations, review articles, opinion papers, reports, and conference papers will all be excluded.

**Search strategy**

The search method will include applying the pre-specified search terms in five selected bibliographic databases. In addition, a grey literature search will be carried out by hand searching the references of selected (review) articles and conference proceedings. Followed by the evaluation of all relevant literature citations found from the search.

**Databases**

The five (5) selected databases to be used include Scopus, MEDLINE, PubMed, CINHAL and Asian Citation Index/Asia Index Medicus. The electronic search strategy will be as follows:

**Library/Database:** Scopus

**Date of Search: 9/12/2021**

**Search String:** (“Epidemiology” OR “Prevalence” OR “Occurrence” OR “Incidence” AND “Multidrug resistant” OR “Multiple drug resistant” OR “Multi-drug resistant” OR “MDR” OR “ESBL” OR “Extended Spectrum Beta Lactamase” OR “Cabapanemase” AND “Klebsiella pneumoniae” OR “K. pneumoniae” OR “Klebsiella infection” AND “Clinical infection” OR “Clinical isolates” OR “Clinical samples” OR “Hospital infection” OR “Hospital-associated infection” OR “Hospital acquired infection” OR “Nosocomial infection” OR “HAI” AND “Indonesia” OR “Cambodia” OR “Vietnam” OR “Singapore” OR “Thailand” OR “Malaysia” OR “PDR Lao” OR “Philippines” OR “Myanmar” OR “Burma” OR “Brunei” OR “Timor-Leste” OR “EastTimor”)

**# Hits: 54**

**Library/Database:** MEDLINE (via EBSCOhost)

**Date of Search: 9/12/2021**

**Search String:** (Epidemiology OR Prevalence OR Occurrence OR Incidence) AND (Multidrug resistant OR Multiple drug resistant OR Multi-drug resistant OR MDR OR ESBL OR Extended Spectrum Beta Lactamase OR Cabapanemase)AND (Klebsiella pneumoniae OR K. pneumoniae OR Klebsiella infection) AND (Clinical infection OR Clinical isolates OR Clinical samples OR Hospital infection OR Hospital-associated infection OR Hospital acquired infection OR Nosocomial infection OR HAI) AND (Indonesia OR Cambodia OR Vietnam OR Singapore OR Thailand OR Malaysia OR PDR Lao OR Philippines OR Myanmar OR Burma OR Brunei OR Timor-Leste OR EastTimor)

**# Hits: 3936**

**Library/Database:** CINHAL complete (via Ebscohost)

**Date of Search: 9/12/2021**

**Search String:** (Epidemiology OR Prevalence OR Occurrence OR Incidence) AND (Multidrug resistant OR Multiple drug resistant OR Multi-drug resistant OR MDR OR ESBL OR Extended Spectrum Beta Lactamase OR Cabapanemase)AND (Klebsiella pneumoniae OR K. pneumoniae OR Klebsiella infection) AND (Clinical infection OR Clinical isolates OR Clinical samples OR Hospital infection OR Hospital-associated infection OR Hospital acquired infection OR Nosocomial infection OR HAI) AND (Indonesia OR Cambodia OR Vietnam OR Singapore OR Thailand OR Malaysia OR PDR Lao OR Philippines OR Myanmar OR Burma OR Brunei OR Timor-Leste OR EastTimor)

**# Hits: 215**

**Library/Database:** Global Index Medicus (Asean Region)

**Date of Search: 13/12/2021**

**Search String (Advance Search):** "Epidemiology" OR "Prevalence" OR "Occurrence" OR "Incidence" AND "Multidrug resistant" OR "Multiple drug resistant" OR "Multi-drug resistant" OR "MDR" OR "ESBL" OR "Extended Spectrum Beta Lactamase" OR "Cabapanemase" AND "Klebsiella pneumoniae" OR "K. pneumoniae" OR "Klebsiella infection" AND "Clinical infection" OR "Clinical isolates" OR "Clinical samples" OR "Hospital infection" OR "Hospital-associated infection" OR "Hospital acquired infection" OR "Nosocomial infection" OR "HAI" AND “Indonesia” OR “Cambodia” OR “Vietnam” OR “Singapore” OR “Thailand” OR “Malaysia” OR “PDR Lao” OR “Philippines” OR “Myanmar” OR “Burma” OR “Brunei” OR “Timor-Leste” OR “EastTimor”

**# Hits: 5**

**Library/Database: PubMed**

**Date of Search: 9/12/2021**

**Search String:** (Epidemiology OR Prevalence OR Occurrence OR Incidence) AND (Multidrug resistant OR Multiple drug resistant OR Multi-drug resistant OR MDR OR ESBL OR Extended Spectrum Beta Lactamase OR Cabapanemase)AND (Klebsiella pneumoniae OR K. pneumoniae OR Klebsiella infection) AND (Clinical infection OR Clinical isolates OR Clinical samples OR Hospital infection OR Hospital-associated infection OR Hospital acquired infection OR Nosocomial infection OR HAI) AND (Indonesia OR Cambodia OR Vietnam OR Singapore OR Thailand OR Malaysia OR PDR Lao OR Philippines OR Myanmar OR Burma OR Brunei OR Timor-Leste OR EastTimor)

**# Hits: 179**

Total # of citations prior to de-duplication: 4389

Total # of citations after de-duplication in Mendeley: 3796

Total # citations added from manual search: 3

Total # citations (de-duplicated + manual search): 3799

**Data selection, collection, and management**

All identified citations will be exported to the Mendeley reference manager for the removal of duplicates after the database search is completed. The de-duplicated citations will then be exported to the Rayyan Intelligent Systematic Review software (Ouzzani et al., 2016) for the title/abstract and full text screening based on the study inclusion and exclusion criteria. Four (4) independent reviewers would undertake the screening process, with a fifth and sixth reviewers deciding on areas of dispute between the four reviewers. The screening process for study selection will be summarised in a PRISMA flow diagram. The data extraction process will start with the creation of a priori data extraction form in a Microsoft Excel (MS) spreadsheet. Afterwards, the characteristics of the studies that will be included, as well as other relevant data, will be retrieved and inputted in the extraction data form. The data that will be extracted include: 1) study characteristics: title, author, country of study, year of publication, and study design; 2) baseline characteristics of study population: sex distribution, sample size, age groups, site of infection; 3) the proportion of MDR *K. pneumoniae*, ESBL *K. pneumoniae*, predominant ESBL genes and harbouring ESBL genes: 4) Screening and confirmatory tests methods. Four independent reviewers will carry out the complete data extraction procedure, which will be validated by a fifth reviewer.

**Outcomes**

Primary outcome: to determine the overall proportion (prevalence) of MDR *Klebsiella pneumoniae* among patients in hospitals

Secondary outcomes: to determine ESBL prevalence, to assess the predominant ESBL occurring genes, assess ESBL harbouring genes, determine sex distribution of ESBL prevalence, assess the site of infection for MDR ESBL *K. pneumoniae* and screening and confirmatory methods adopted in hospitals for the isolation of MDR ESBL *K. pneumoniae*.

**Risk of Bias (quality) assessment**

All articles to be included will be subjected to a quality assessment using the Joanna Briggs Institute critical appraisal checklist for studies reporting prevalence data (Institute, 2011). The appraisal tool has 9 questions that will be answered either; Yes (Y), No (N), Unclear (UC) or Not applicable (NA). Scores will be awarded as; Y = 1, N = 0, UC = 0, and NA will attract no score. Based on the scores the quality of the studies will be graded; studies with ≤50% scores will be deemed low quality studies. Those with >50% - 69% will be termed moderate quality studies. While high quality studies will be those with ≥70% scores. The quality assessment process will be conducted by four independent reviewers and two other reviewers would verify.

**Meta-analysis**

**Statistical Assessment**

MetaXL software (add-in for Microsoft Excel) will be used for quantitative analysis of the extracted data. The meta-analysis and pooling of the prevalence estimate (with the 95% confidence interval) will be done using the quality effect (QE) model and by employing (the transformed) double arcsine method.

Assessment of Heterogeneity

Estimation of statistical heterogeneity amongst the included studies will be done using the X^2^

test Cochrane Q, and I^2^ statistics. An I^2^ value of 0 to ≤40% will be considered as low heterogeneity, >40% to 60% will be regarded as moderate heterogeneity, >60% to 75% will be considered substantial heterogeneity, and >75% to 100% will be considered high heterogeneity.

Publication bias

Provided the number of included studies for the meta-analysis is ≥10, a funnel plot will be constructed to exam for publication bias. In the event of an observed asymmetry on the funnel plot, a further assessment using the Doi plot to evaluate the symmetry of the funnel plot will be carried out. Also, Egger’s regression test will be conducted to test the significance of the asymmetry.

**Sensitivity Analysis**

Sensitivity analysis will be done based on leave one out model to identify the studies that greatly influence the result of the meta-analysis.

**Subgroup Analysis and meta-regression**

Subgroup analysis and meta-regression will be conducted to identify the moderators of heterogeneity in the included studies. The factors to be used in the subgroup analysis will include country (location) of study, ASEAN country subdivision, year of study publication, study sample size, detection method (ESBL screening and confirmatory tests) used, study quality and weight of study in meta-analysis. In the case of meta-regression, factors with considerable heterogeneity in the subgroup analysis will be used in the univariate analysis. Factors with significant univariate analysis will be used subsequently in the multivariate analysis. Due to the low statistical power of the meta-regression 0.25 will be considered as the significant *p* value.

**References**

Abla, H.-H., Chafia, B., Abdesselam, L., Houcine, L., Kaddour, B., & Farida, S. (2016). Multidrug-resistant bacteria isolated from patients hospitalized in Intensive Care Unit in University Hospital of Constantine, Algeria (2011 - 2015). *African Journal of Microbiology Research*, *10*(33), 1328–1336. https://doi.org/10.5897/ajmr2016.8257

Aung, M. S., Win, N. C., San, N., Hlaing, M. S., Myint, Y. Y., Thu, P. P., Aung, M. T., Yaa, K. T., Maw, W. W., Urushibara, N., & Kobayashi, N. (2021). Prevalence of Extended-Spectrum Beta-Lactamase/Carbapenemase Genes and Quinolone-Resistance Determinants in Klebsiella pneumoniae Clinical Isolates from Respiratory Infections in Myanmar. *Microbial Drug Resistance (Larchmont, N.Y.)*, *27*(1), 36–43. https://doi.org/10.1089/mdr.2019.0490

Institute, J. B. (2011). Joanna Briggs Institute Critical Appraisal Checklist for Studies Reporting Prevalence Data. *Adelaide: Joanna Briggs Institute*.

Moher, D., Stewart, L., & Shekelle, P. (2016). Implementing PRISMA-P: recommendations for prospective authors. *Systematic Reviews*, *5*(1), 1–2.

Morrison, L., & Zembower, T. R. (2020). Antimicrobial Resistance. *Gastrointestinal Endoscopy Clinics of North America*, *30*(4), 619–635. https://doi.org/10.1016/j.giec.2020.06.004

Naeemmudeen, N. M., Mohd Ghazali, N. A. N., Bahari, H., Ibrahim, R., Samsudin, A. D., & Jasni, A. S. (2021). Trends in antimicrobial resistance in Malaysia. *The Medical Journal of Malaysia*, *76*(5), 698–705. https://search.ebscohost.com/login.aspx?direct=true&db=mdc&AN=34508377&site=ehost-live

Nguyen, T. N. T., Nguyen, P. L. N., Le, N. T. Q., Nguyen, L. P. H., Duong, T. B., Ho, N. D. T., Nguyen, Q. P. N., Pham, T. D., Tran, A. T., The, H. C., Nguyen, H. H., Nguyen, C. V. V., Thwaites, G. E., Rabaa, M. A., & Pham, D. T. (2021). Emerging carbapenem-resistant Klebsiella pneumoniae sequence type 16 causing multiple outbreaks in a tertiary hospital in southern Vietnam. *Microbial Genomics*, *7*(3). https://doi.org/10.1099/mgen.0.000519

Ouzzani, M., Hammady, H., Fedorowicz, Z., & Elmagarmid, A. (2016). Rayyan-a web and mobile app for systematic reviews. *Systematic Reviews*, *5*(1), 1–10. https://doi.org/10.1186/s13643-016-0384-4

Tanwar, J., Das, S., Fatima, Z., & Hameed, S. (2014). Multidrug resistance: An emerging crisis. *Interdisciplinary Perspectives on Infectious Diseases*, *2014*(July). https://doi.org/10.1155/2014/541340

Yamasaki, S., Shigemura, K., Osawa, K., Kitagawa, K., Ishii, A., Kuntaman, K., Shirakawa, T., Miyara, T., & Fujisawa, M. (2021). Genetic analysis of ESBL-producing Klebsiella pneumoniae isolated from UTI patients in Indonesia. *Journal of Infection and Chemotherapy : Official Journal of the Japan Society of Chemotherapy*, *27*(1), 55–61. https://doi.org/10.1016/j.jiac.2020.08.007
