# Supplementary material for: Epidemiology of multidrug-resistant Klebsiella pneumoniae infection in clinical setting in South-Eastern Asia: a systematic review and meta-analysis
Source: Antimicrob Resist Infect Control. 2023 Dec 7;12:142. doi: 10.1186/s13756-023-01346-5 (PMC10704709; doi:10.1186/s13756-023-01346-5)
Supplement: Supplementary file 1 — Additional file 1: Figures S1. Subgroups analysis forest plots. File S1. PRISMA 2020 checklist. File S2. PRISMA-P 2015 checklist. File S3. Study protocol. File S4. De-duplicated citations. File S5. Included studies. Table S2. JBI critical appraisal checklist. Table S3. ESBL summary. [file 13756_2023_1346_MOESM1_ESM.zip › Supplementary materials/S5 File List of included studie1.docx]

**List of included studies**

Al-Marzooq, F., Mohd Yusof, M. Y., & Tay, S. T. (2015). Molecular Analysis of Antibiotic Resistance Determinants and Plasmids in Malaysian Isolates of Multidrug Resistant Klebsiella pneumoniae. *PloS One*, *10*(7), e0133654. https://doi.org/10.1371/journal.pone.0133654

Apisarnthanarak, A., Kiratisin, P., & Mundy, L. M. (2008). Clinical and molecular epidemiology of healthcare-associated infections due to extended-spectrum beta-lactamase (ESBL)-producing strains of Escherichia coli and Klebsiella pneumoniae that harbor multiple ESBL genes. *Infection Control and Hospital Epidemiology*, *29*(11), 1026–1034. https://doi.org/10.1086/591864

Aung, M. S., Win, N. C., San, N., Hlaing, M. S., Myint, Y. Y., Thu, P. P., Aung, M. T., Yaa, K. T., Maw, W. W., Urushibara, N., & Kobayashi, N. (2021). Prevalence of Extended-Spectrum Beta-Lactamase/Carbapenemase Genes and Quinolone-Resistance Determinants in Klebsiella pneumoniae Clinical Isolates from Respiratory Infections in Myanmar. *Microbial Drug Resistance (Larchmont, N.Y.)*, *27*(1), 36–43. https://doi.org/10.1089/mdr.2019.0490

Berglund, B., Hoang, N. T. B., Tärnberg, M., Le, N. K., Nilsson, M., Khu, D. T. K., Svartström, O., Welander, J., Nilsson, L. E., Olson, L., Dien, T. M., Le, H. T., Larsson, M., & Hanberger, H. (2019). Molecular and phenotypic characterization of clinical isolates belonging to a KPC-2-producing strain of ST15 Klebsiella pneumoniae from a Vietnamese pediatric hospital. *Antimicrobial Resistance and Infection Control*, *8*, 156. https://doi.org/10.1186/s13756-019-0613-4

Kiratisin, P., Apisarnthanarak, A., Laesripa, C., & Saifon, P. (2008). Molecular characterization and epidemiology of extended-spectrum-beta-lactamase-producing Escherichia coli and Klebsiella pneumoniae isolates causing health care-associated infection in Thailand, where the CTX-M family is endemic. *Antimicrobial Agents and Chemotherapy*, *52*(8), 2818–2824. https://doi.org/10.1128/AAC.00171-08

Kiratisin, P., Apisarnthanarak, A., Saifon, P., Laesripa, C., Kitphati, R., & Mundy, L. M. (2007). The emergence of a novel ceftazidime-resistant CTX-M extended-spectrum beta-lactamase, CTX-M-55, in both community-onset and hospital-acquired infections in Thailand. *Diagnostic Microbiology and Infectious Disease*, *58*(3), 349–355. https://doi.org/10.1016/j.diagmicrobio.2007.02.005

Kusum, M., Wongwanich, S., Dhiraputra, C., Pongpech, P., & Naenna, P. (2004). Occurrence of extended-spectrum beta-lactamase in clinical isolates of Klebsiella pneumoniae in a University Hospital, Thailand. *Journal of the Medical Association of Thailand = Chotmaihet Thangphaet*, *87*(9), 1029–1033. https://search.ebscohost.com/login.aspx?direct=true&db=mdc&AN=15516002&site=ehost-live

Lee, Y. Q., Ahmad Kamar, A., Velayuthan, R. D., Chong, C. W., & Teh, C. S. J. (2021). Clonal relatedness in the acquisition of intestinal carriage and transmission of multidrug resistant (MDR) Klebsiella pneumoniae and Escherichia coli and its risk factors among preterm infants admitted to the neonatal intensive care unit (NICU). *Pediatrics and Neonatology*, *62*(2), 129–137. https://doi.org/10.1016/j.pedneo.2020.10.002

Lim, K. T., Yeo, C. C., Md Yasin, R., Balan, G., & Thong, K. L. (2009). Characterization of multidrug-resistant and extended-spectrum beta-lactamase-producing Klebsiella pneumoniae strains from Malaysian hospitals. *Journal of Medical Microbiology*, *58*(Pt 11), 1463–1469. https://doi.org/10.1099/jmm.0.011114-0

Low, Y.-M., Yap, P. S.-X., Abdul Jabar, K., Ponnampalavanar, S., Karunakaran, R., Velayuthan, R., Chong, C.-W., Abu Bakar, S., Md Yusof, M. Y., & Teh, C. S.-J. (2017). The emergence of carbapenem resistant Klebsiella pneumoniae in Malaysia: correlation between microbiological trends with host characteristics and clinical factors. *Antimicrobial Resistance and Infection Control*, *6*, 5. https://doi.org/10.1186/s13756-016-0164-x

Mobasseri, G., Thong, K. L., Rajasekaram, G., & Teh, C. S. J. (2020). Molecular characterization of extended-spectrum β-lactamase-producing Klebsiella pneumoniae from a Malaysian hospital. *Brazilian Journal of Microbiology : [Publication of the Brazilian Society for Microbiology]*, *51*(1), 189–195. https://doi.org/10.1007/s42770-019-00208-w

Mohd Helmi, U., Mohd Desa, M. N., Taib, N. M., Tengku Jamaluddin, T. Z. M., & Masri, S. N. (2016). Multiple ambler class A ESBL genes among Klebsiella pneumoniae isolates in a Malaysian district hospital. *Tropical Biomedicine*, *33*(1), 109–119. https://search.ebscohost.com/login.aspx?direct=true&db=mdc&AN=33579148&site=ehost-live

Niumsup, P. R., Tansawai, U., Boonkerd, N., Polwichai, P., & Dejsirilert, S. (2008). Dissemination of extended-spectrum beta-lactamase-producing Klebsiella pneumoniae and Escherichia coli in Thai hospitals. *Journal of Infection and Chemotherapy : Official Journal of the Japan Society of Chemotherapy*, *14*(6), 404–408. https://doi.org/10.1007/s10156-008-0642-z

Saharman, Y. R., Karuniawati, A., Sedono, R., Aditianingsih, D., Goessens, W. H. F., Klaassen, C. H. W., Verbrugh, H. A., & Severin, J. A. (2020). Clinical impact of endemic NDM-producing Klebsiella pneumoniae in intensive care units of the national referral hospital in Jakarta, Indonesia. *Antimicrobial Resistance and Infection Control*, *9*(1), 61. <https://doi.org/10.1186/s13756-020-00716-7>

Severin, J. A., Mertaniasih, N. M., Kuntaman, K., Lestari, E. S., Purwanta, M., Lemmens-Den Toom, N., Duerink, D. O., Hadi, U., van Belkum, A., Verbrugh, H. A., Goessens, W. H., JA, S., NM, M., Kuntaman, K., ES, L., Purwanta, M., N, L.-D. T., DO, D., Hadi, U., … WH, G. (2010). Molecular characterization of extended-spectrum beta-lactamases in clinical Escherichia coli and Klebsiella pneumoniae isolates from Surabaya, Indonesia. *The Journal of Antimicrobial Chemotherapy*, *65*(3), 465–469. https://doi.org/10.1093/jac/dkp471

Sianipar, O., Asmara, W., Dwiprahasto, I., & Mulyono, B. (2019). Mortality risk of bloodstream infection caused by either Escherichia coli or Klebsiella pneumoniae producing extended-spectrum β-lactamase: a prospective cohort study. *BMC Research Notes*, *12*(1), 719. https://doi.org/10.1186/s13104-019-4751-9

Tiongco, R. E., Arceo, E., Dizon, D., Navarro, A., Rivera, N., Salita, C., & Singian, E. (2018). Phenotypic evaluation of ESBL- and carbapenemaseproducing Escherichia coli and Klebsiella pneumoniae from a teaching hospital in the Philippines. *Tropical Biomedicine*, *35*(4), 1064–1074. https://search.ebscohost.com/login.aspx?direct=true&db=mdc&AN=33601853&site=ehost-live

Trang, N. H. T., Nga, T. V. T., Campbell, J. I., Hiep, N. T., Farrar, J., Baker, S., & Duy, P. T. (2013). The characterization of ESBL genes in Escherichia coli and Klebsiella pneumoniae causing nosocomial infections in Vietnam. *Journal of Infection in Developing Countries*, *7*(12), 922–928. https://doi.org/10.3855/jidc.2938

van Aartsen, J. J., Moore, C. E., Parry, C. M., Turner, P., Phot, N., Mao, S., Suy, K., Davies, T., Giess, A., Sheppard, A. E., Peto, T. E. A., Day, N. P. J., Crook, D. W., Walker, A. S., & Stoesser, N. (2019). Epidemiology of paediatric gastrointestinal colonisation by extended spectrum cephalosporin-resistant Escherichia coli and Klebsiella pneumoniae isolates in north-west Cambodia. *BMC Microbiology*, *19*(1), 59. https://doi.org/10.1186/s12866-019-1431-9

Yamasaki, S., Shigemura, K., Osawa, K., Kitagawa, K., Ishii, A., Kuntaman, K., Shirakawa, T., Miyara, T., & Fujisawa, M. (2021). Genetic analysis of ESBL-producing Klebsiella pneumoniae isolated from UTI patients in Indonesia. *Journal of Infection and Chemotherapy : Official Journal of the Japan Society of Chemotherapy*, *27*(1), 55–61. https://doi.org/10.1016/j.jiac.2020.08.007

Zheng, S., Tan, S. Y., & Oh, H. M. (2019). Klebsiella Pneumoniae Visceral Organ Abscesses - Clinical Characteristics. *Annals of the Academy of Medicine, Singapore*, *48*(2), 48–54. https://search.ebscohost.com/login.aspx?direct=true&db=mdc&AN=30926976&site=ehost-live
